# Drug‐induced pneumonitis risk in diffuse large B‐cell/follicular lymphoma patients treated with R‐CHOP‐like regimen is associated with the use of granulocyte colony‐stimulating growth factors

**DOI:** 10.1002/cam4.6898

**Published:** 2024-01-01

**Authors:** Elina Kaprio, Roosa Prusila, Susanna Tokola, Milla E. L. Kuusisto, Esa Jantunen, Hanne Kuitunen, Taina Turpeenniemi‐Hujanen, Outi Kuittinen

**Affiliations:** ^1^ Faculty of Health Medicine, Institute of Clinical Medicine University of Eastern Finland Kuopio Finland; ^2^ Department of Pediatrics Kuopio University Hospital Kuopio Finland; ^3^ Department of Oncology and Radiotherapy, Medical Research Center Oulu University Hospital Oulu Finland; ^4^ Translational Medicine Research Unit University of Oulu Oulu Finland; ^5^ The North Karelia Central Hospital Joensuu Finland; ^6^ Cancer Center, Kuopio University Hospital Kuopio Finland; ^7^ Länsi‐Pohja Central Hospital, Kauppakatu 25 Kemi Finland

**Keywords:** diffuse large B‐cell lymphoma, drug‐induced pneumonitis, follicular lymphoma, granulocyte colony‐stimulating factors

## Abstract

**Background:**

Rituximab‐based combinations are the standard of care in diffuse large B‐cell lymphoma (DLBCL) and follicular lymphoma (FL). Despite being on market for over 20 years, some of the adverse effects associated with the use of rituximab are not well known. Drug‐induced interstitial pneumonitis (DIP) is a potentially fatal complication of the treatment. Granulocyte colony‐stimulating factors (G‐CSF) are supportive agents commonly used to prevent neutropenic infections. G‐CSF are reported to have pulmonary toxicity, but the risk of DIP is greater when used in combination with other potentially pulmotoxic agents.

**Methods:**

In this retrospective study, we reported the G‐CSF use and risk of DIP in 234 DLBCL patients and 87 FL patients receiving R‐CHOP‐type immunochemotherapy.

**Results:**

In 72% of patients, the treatment included a G‐CSF support. The overall incidence of treatment‐induced pneumonitis was 6.9% in this patient group. All the DIP cases (*n* = 16) were among patients receiving G‐CSF support (*p* = 0.03). Older age (over 60 years) and higher disease stage (Ann Arbor 3–4) also increased the risk of DIP.

**Conclusions:**

These findings suggest that the use of G‐CSF increases the risk of DIP, when used in combination with rituximab‐containing regimen.

## Graphical abstract

Our findings suggest that the use of granulocyte colony‐stimulating factors increases the incidence of drug‐induced pneumonitis in patients with diffuse large cell lymphoma and follicular lymphoma receiving R‐CHOP‐type treatment.

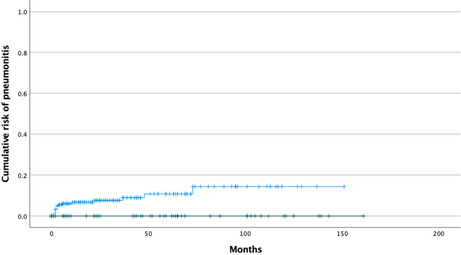

## 1 INTRODUCTION

Diffuse large B‐cell lymphoma (DLBCL) and follicular lymphoma (FL) stand as the common types of non‐Hodgkin lymphomas in the Western world, accounting for 25%–58% and 20%–40% of all non‐Hodgkin lymphoma cases, respectively.^1^, ^2^, ^3^, ^4^ Annually in Finland approximately 600 new DLBCL cases and 300 new FL cases are being diagnosed.^5^

Rituximab, a CD20 antibody, presently constitutes an integral part of the standard therapeutic protocol for both DLBCL and FL. The integration of rituximab has improved outcomes for both DLBCL and FL, resulting improved progression‐free survival and overall survival rates.^6^, ^7^, ^8^ From a broader perspective, rituximab is typified by favorable tolerability profile. However, cases of rituximab‐induced pneumonitis have been documented,^9^, ^10^ although the precise frequency remains unclear. The manufacturer suggests the incidence of rituximab‐induced pneumonitis to be less than 1 per 10,000 cases.^11^

Granulocyte colony‐stimulating factors (G‐CSF) are commonly used as supportive agents to reduce the risk of neutropenic infections and sustain chemotherapy dose‐intensity.^12^ The use of G‐CSF is recommended when the cumulative risk of febrile neutropenia (FN) reaches or exceeds 20%.^13^, ^14^ FN can potentially be life‐threatening complication of immunochemotherapy treatment. FN incidence related to R‐CHOP‐type treatment is reported to be around 20% in different studies.^14^, ^15^, ^16^

Paradoxically, often the potential adverse effects of G‐CSF use have not been considered. The use of filgrastim and pegfilgrastim can cause pulmonary adverse reactions such as interstitial pneumonitis and even ARDS, the incidence being uncommon varying from >1/1000 to <1/100.^17^, ^18^ Select studies have shown an elevated likelihood for DIP in DLBCL patients when G‐CSF is administered concurrently with rituximab.^19^, ^20^ However, no analogous studies have been conducted in the context of FL patients.

In the treatment of Hodgkin lymphoma, the bleomycin‐induced pulmonary toxicity is a well‐recognized complication.^21^, ^22^, ^23^ It has been suspected that G‐CSF administration during ABVD (doxorubicin, bleomycin, vinblastine, dacarbazine) regimens could heighten the incidence of bleomycin‐induced pneumonitis, although findings have been conflicting.^24^, ^25^

In this retrospective analysis, our intent was to find out the incidence of DIP among patients undergoing R‐CHOP‐type chemotherapy for DLBCL and FL. Furthermore, we sought to investigate whether concurrent administration of G‐CSF increases this risk. As a secondary objective, we aimed to assess whether confirmed or suspected DIP had influence on the patient's overall survival.

## 2 MATERIALS AND METHODS

### 2.1 Patient selection, staging, and treatment

This retrospective registry study included patients diagnosed with diffuse large b‐cell lymphoma (DLBCL) and follicular lymphoma (FL) who received treatment involving R‐CHOP‐type immunochemotherapy. The patient data was collected from the medical records of Oulu University Hospital and North Karelia Central Hospital in Finland. The data included 234 DLBCL patients and 87 FL patients, all of whom had been diagnosed between the years 1999 and 2017.

All patients enrolled in the study underwent first line immunochemotherapy utilizing the R‐CHOP‐type (R‐CHOP, R‐CHOEP, R‐CEOP, or R‐CVOP) regimen. The R‐CHOP regimen consists of rituximab combined with cyclophosphamide, doxorubicin, vincristine, and prednisone and the treatment can be altered with etoposide or vinblastine. The treatment cycles ranged from 1 to 8 instances. Patients who had received intravenous high‐dose methotrexate for central nervous system prophylaxis were excluded from the study.

This retrospective registry study was performed in accordance with the Declaration of Helsinki, and its conduct conformed to the relevant Finnish ethical and legal frameworks. According to Finnish laws and legislations ethical committee approval is not needed for registry studies. Instead registry studies, which do not interfere with patients treatment and the patients are not contacted, are approved by institutions authorities. The retrospective and non‐interventional character of the study negated the necessity for informed consent in compliance with national legislation.

Baseline clinical attributes, inclusive of disease staging in accordance with the Ann Arbor system,^26^ the International Prognostic Index (IPI), and the follicular lymphoma prognostic index (FLIPI),^27^, ^28^ were documented. The administration of granulocyte colony‐stimulating factor (G‐CSF) during treatment was recorded, wherein patients qualified as a G‐CSF users if they were subjected to this growth factor in at least one treatment cycle. The application of pegfilgrastim or daily filgrastim for approximately 7 days represented the protocols for G‐CSF utilization. The initiation of G‐CSF occurred at the earliest 24 h after the last chemotherapy infusion.

### 2.2 Pneumonitis characteristics

In cases wherein patients had undergone hospitalization due to symptoms suggestive of pneumonia, more detailed information such as possible pneumonitis symptoms, results of the CT‐scans, possible lung examinations (bronchoalveolar lavage, spirometry, diffusion capacity) and findings in bacterial, fungal, and viral studies, as well as the response to antimicrobial and steroid treatment was collected in order to find the possible pneumonitis cases.

The diagnostic criteria for pneumonitis were:

1. Radiological findings indicative of pneumonitis, exemplified by manifestations like ground glass opacity, diffuse lung infiltrates, tree‐in‐bud patterns, and mosaic attenuation.
2. Negative outcomes in bacterial cultures, viral polymerase chain reaction (PCR), immunohistochemistry evaluations for pneumocystis carinii, and fungal cultures during bronchoalveolar lavage.
3. Concurrent presentation of symptoms such as cough, dyspnea, and fever.
4. Ineffectiveness of broad‐spectrum antibiotic therapy.
5. Demonstrated efficacy of corticosteroid treatment.

While diminished diffusion capacity and spirometry parameters provided additional support for diagnosing pneumonitis, they were not included in the diagnostic criteria. Some of the pneumonitis cases were regarded as suspected cases if the patient had also received treatment for pneumocystis infection but pneumocystis staining and PCR in bronchoalveolar lavage were negative.

In all identified cases, the therapeutic approach to pneumonitis involved the administration of high‐dose oral corticosteroid prednisolone, with a median daily dose of 1 mg/kg.

### 2.3 Statistical analysis

The primary objective was to evaluate the incidence of DIP and to compare the DIP incidence between groups receiving treatment with or without G‐CSF support. Also, other clinical features associated with the risk were analyzed. The secondary objective was to evaluate if definitive or suspected pneumonitis had effect on patients' overall survival. Categorical variables underwent analysis employing the two‐sided Pearson chi‐squared test or Fisher's exact test as appropriate. Continuous variables were analyzed using the Mann–Whitney *U* test or Kruskal–Wallis test. Survival analyses with corresponding *p*‐values were calculated using the Kaplan–Meier method with the log‐rank test. IBM SPSS 27 was used for statistical analysis and *p*‐values <0.05 were considered to be significant.

## 3 RESULTS

### 3.1 Patient demographics

The baseline demographic and clinical characteristics of all 321 patients according to diagnosis of pneumonitis are listed in Table 1. The male/female ratio was 1.2 and overall median age was 69 ± 13 years (range: 19–96). A little more than half (53%) of the patients had an advanced stage disease. 50% of patients diagnosed with FL had FLIPI ≥3, and 60% of patients with DLBCL had IPI 3–5.

**TABLE 1:** Patient demographics.

	DRUG‐INDUCED PNEUMONITIS		*p*‐Value
	yes (*n* = 16)	no (*n* = 305)	
Diagnosis			
DLBCL	12 (75%)	222 (73%)	0.846
FL	4 (25%)	83 (27%)	
Gender			
Male	8 (50%)	165 (54%)	0.932
Female	7 (44%)	138 (45%)	
Missing	1 (6%)	2 (1%)	
Age			
<60 years	2 (10%)	113 (37%)	0.046
>60 years	14 (90%)	192 (63%)	
Missing	0 (0)	0 (0)	
Stage			
1–2	2 (10%)	142 (47%)	0.004
3–4	14 (90%)	148 (49%)	
Missing	0 (0)	15 (4%)	
IPI‐score (DLBCL)			
0–2	6 (50%)	127 (57%)	0.471
3–5	6 (50%)	83 (37%)	
Missing	0 (0)	12 (6%)	
FLIPI‐score (FL)			
0–2	2 (50%)	51 (61%)	0.578
3–5	2 (50%)	29 (35%)	
Missing	0 (0)	3 (4%)	
G‐CSF use			
Yes	16 (100%)	216 (70%)	0.030
No	0 (0)	65 (21%)	
Missing	0 (0)	24 (9%)	

Abbreviations: DLBCL, diffuse large B‐cell lymphoma; FL, follicular lymphoma; FLIPI, follicular lymphoma prognostic index; G‐CSF, Granulocyte colony‐stimulating factor; IPI, International Prognostic Index.

### 3.2 Association of growth factor use with drug‐induced pneumonitis

The majority, 159 of the DLBCL‐patients, had received treatment with growth factor support and 52 were treated without growth factor support. The information considering G‐CSF‐use was missing from 23 patients. Ten patients had definitive and two suspected DIP; all these patients had received G‐CSF support. This difference was statistically significant, *p* = 0.041.

Also, most (73) FL patients received the treatment with growth factor support and 13 were treated without it, the information about G‐CSF use was missing from 1 patient. In FL population there was three definitive and one suspected case of DIP. All patients suffering from DIP had received G‐CSF. This difference was not statistically significant *p* = 0.387.

**FIGURE 1:**
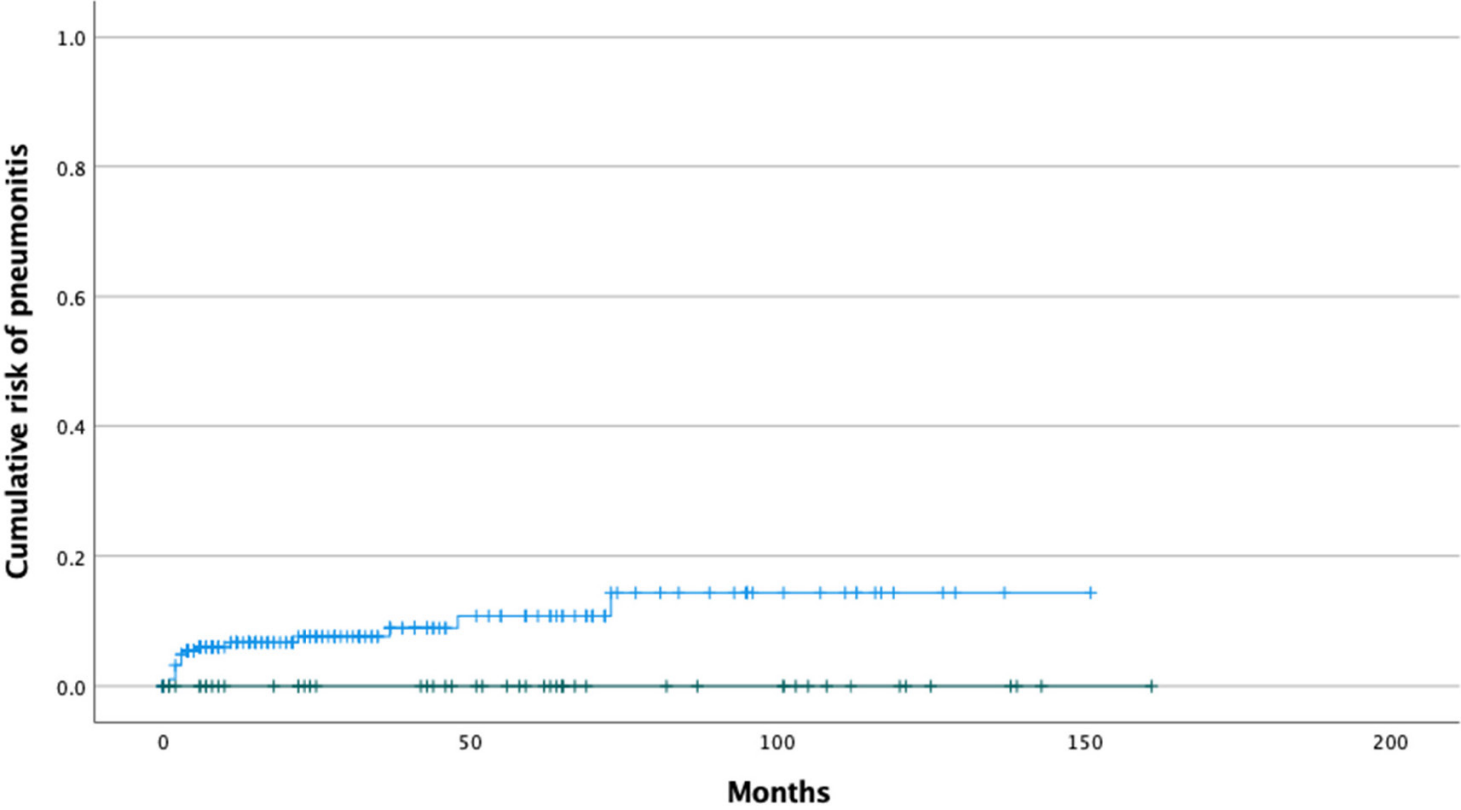
Kaplan–Meier estimate of risk of definitive and suspected DIP according to the use of GCS‐F: patients receiving growth factor support in blue and patients with no growth factor support in green.

The overall risk of definitive and suspected DIP was 5,0% for all patients. After combining both diagnosis groups 13 patients (5,6%) that had received G‐CSF had definitive drug‐induced pneumonitis and 3 patients had suspected DIP. When combining definitive and suspected DIP cases to one group (*n* = 16), it counted for 6.9 percent of all patients receiving G‐CSF (Figure 1). This difference comparing to patients not receiving G‐CSF was statistically significant *p* = 0.030; the 95% confidence interval for the 6.9% risk difference was 3.6%–10.1%.

### 3.3 Other risk factors

Risk of DIP did not correlate with patient's gender, grade of the lymphoma in FL, B‐symptoms or patients pre‐existing autoimmune disease. Most of the DIP occurred in patients, who were 60 years or older, when only two patients younger than 60 had DIP; the risk for older patients was 4.2 fold (95% confidence interval 0.96 to 17.9, Fisher's exact *p*‐value 0.036). Most of the patients with DIP had stage 3 or 4 lymphoma, risk ratio being 6.2 (95% CI 1.43 to 26.7). This difference was statistically significant *p* = 0.044 (Fisher exact test 0.0042).

### 3.4 DIP and survival outcome

**FIGURE 2:**
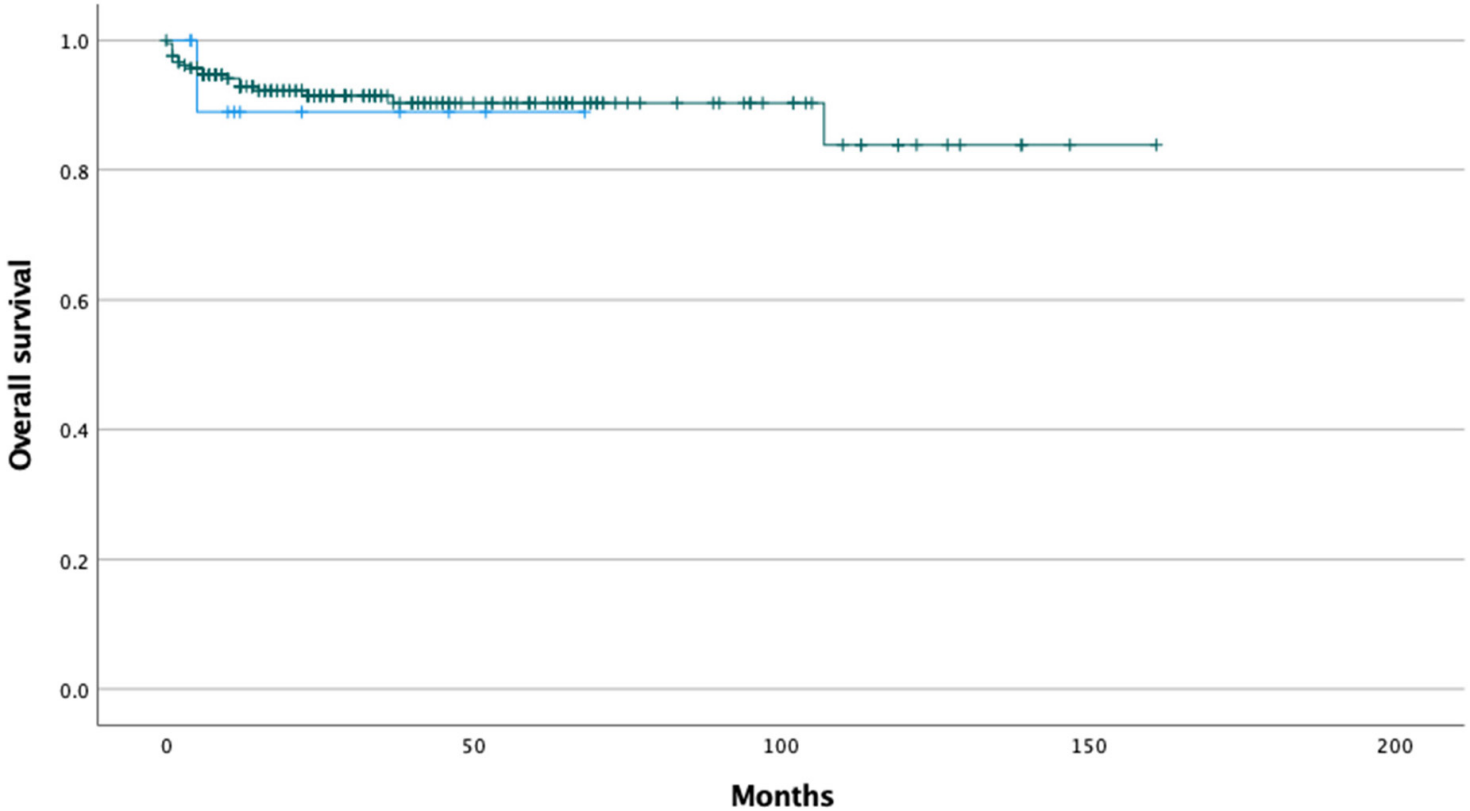
Kaplan–Meier estimate of overall survival of patients diagnosed with DLBCL according to pneumonitis status, patients with definitive or suspected pneumonitis in blue, and patients with no pneumonitis in green. (*p* = 0.840).

**FIGURE 3:**
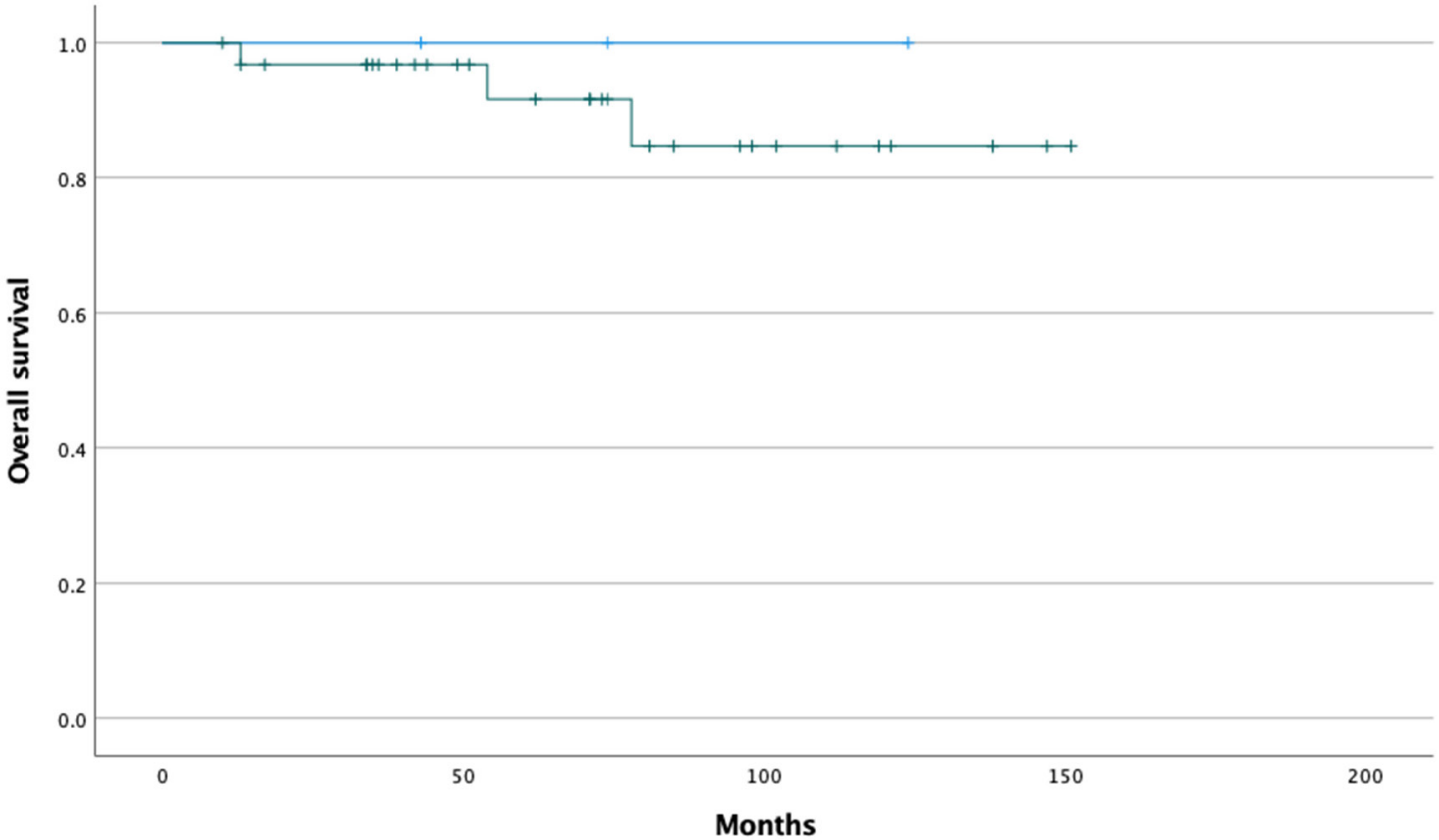
Kaplan–Meier estimate of overall survival of patients diagnosed with FL according to pneumonitis status, patients with definitive or suspected pneumonitis in blue, and patients with no pneumonitis in green. (*p* = 0.597).

The incidence of DIP was highest after second or fourth immunochemotherapy cycle with variation from 1 to 6 cycles. The median follow‐up time was 34 months. DIP did not affect patients' survival outcome (Figure 2 and Figure 3). One patient with DIP developed acute respiratory distress syndrome with lethal outcome. Other 15 patients with either definitive or suspected DIP recovered.

All the patients received per oral corticosteroids as their treatment for pneumonitis with median dose of prednisolone 1 mg/kg. In many cases, rituximab and G‐CSF was discontinued after the DIP diagnosis and the treatment was continued with chemotherapy only if needed.

## 4 DISCUSSION

This article describes the correlation between the utilization of G‐CSF with the risk of drug‐induced pneumonitis among B‐cell lymphoma patients receiving R‐CHOP‐type immunochemotherapy. Within the whole study population, the incidence of DIP was noted to be 5%, exclusively observed within individuals receiving G‐CSF support. Among patients receiving G‐CSF 6.9% were diagnosed with definitive or suspected DIP. These findings indicate that administration of G‐CSF during R‐CHOP‐type treatment augments the risk of DIP. This observation finds resonance with certain Asian studies, which have indicated that the use of rituximab and G‐CSF in combination with CHOP‐type chemotherapy is a contributing factor to interstitial pneumonitis.^19^, ^20^ We propose that combination of rituximab and G‐CSF increases the risk of DIP.

Rituximab, a monoclonal antibody targeting CD20 antigens on B‐cells, operates through mechanisms encompassing enhancement of cell‐mediated immunity, complement activation, and induction of apoptosis via intracellular signaling. Its use has highly increased since the early 21st century, showing efficacy in improving outcomes for DLBCL and FL, in both the terms of progression‐free and overall survival.^6^, ^7^, ^8^ Consequently, rituximab is now integral to the standard care regimen for B‐cell lymphomas.

Drug‐induced pneumonitis, an infrequent yet potentially fatal complication associated with rituximab therapy, is examined in this context. While isolated case reports and articles discuss rituximab‐induced pneumonitis,^9^, ^10^, ^29^, ^30^ a comprehensive understanding of its incidence remains unclear, despite the long history of rituximab usage.

In this study strict diagnostic criteria for classifying drug‐induced pneumonitis were used, mandating pulmonary symptoms (fever, cough, dyspnea), radiological findings (diffuse lung infiltrates and/or ground glass opacity), negative microbiologic assays. It was also required that treatment with broad‐spectrum antibiotics did not improve symptoms or laboratory parameters, but oral administration of corticosteroid was effective. Some cases, characterized by simultaneous pneumocystis carinii treatment, were categorized as suspected pneumonitis cases, when immunohistochemistry of pneumocystis carinii was negative.

Clinical diagnosis of DIP predominantly relies on typical radiological findings and exclusion of alternative etiologies, primarily infectious. A special challenge is differential diagnosis with pneumocystis jirovecii (PCJ). Asymptomatic PCJ colonization in lung is common even among healthy individuals.^31^ Earlier immunohistochemistry‐based methods were less sensitive for this kind of asymptomatic colonization. After adopting the highly sensitive polymerase chain reaction (PCR) technology, this challenge in differential diagnostic has multiplied especially because PCR is able to detect also asymptomatic colonization without any causality to patients' symptoms.^32^ This implies the importance of the development of quantitative PCR methodologies to discriminate clinical infection from asymptomatic colonization. In the current context of PCR testing our hypothesis is that a proportion of DIP may be misdiagnosed to PCJ.

Case documentation exists linking G‐CSF to DIP as single agent the manufacturer suggests the incidence of G‐CSF associated DIP to be from 0,1% to 1% for pegfilgrastim and filgrastim.^17^, ^18^, ^33^ The reported DIP risk associated with G‐CSF use is therefore higher than the risk of DIP associated with rituximab. Furthermore, G‐CSF escalates pulmonary toxicity risk in synergy with other pneumotoxic agents; for instance, its role in Hodgkin's lymphoma treatment is suspected to be associated with higher bleomycin‐induced lung toxicity risk.^24^ These drug‐induced pneumonitis diseases are often serious cases with distressing symptoms from which the recovery may take days, weeks, or even months, and pneumonitis can even be potentially fatal. Notably, our study demonstrated a higher incidence of DIP when rituximab‐containing regimen were used in combination with G‐CSF demonstrating an additional pneumotoxic effect between rituximab and G‐CSF.

G‐CSF is typically advised for conditions involving a 20% or greater risk of febrile neutropenia. G‐CSF is, however, widely used, outside this indication also, presumably because it considered to be safe. G‐CSF's use induces also other adverse effects beyond DIP.^33^ Typical acute adverse effects can appear as bone pain, headache, and fatigue, while long‐term complications may include elevated leukemia risk.^34^, ^35^, ^36^ Thus, we would like to underline that, when considering using G‐CSF support, in order to balance the risk benefit ratio, it is important to follow the guidelines^13^, ^14^ and to use the correct dosing and timing of G‐CSF support.

In this study the G‐CSF used was either pegfilgrastim administered once 24–48 h after the end of chemotherapy or filgrastim administered daily for approximately 7 days. The 7 days schedule for filgrastim is based on studies, demonstrating its better ability to prevent FN better compared to shorter courses.^37^, ^38^

The study identifies two patient cohorts with substantially elevated drug‐induced pneumonitis risk: those aged over 60 (4‐fold risk) and those at advanced lymphoma stages (6‐fold risk). G‐CSF use is more prevalent among elderly and high‐tumor‐mass patients, groups vulnerable to treatment complications and febrile neutropenia. We strongly suggest that the association of G‐CSF, and rituximab is increasing the risk for drug‐induced pneumonitis. However, in the present work, we cannot fully exclude the possibility that differences in age and stage may have affected the observed associations between DIP with the G‐CSF use.

The study's strengths are strict diagnostic criteria, comprehensive imaging, bronchoscopy, and a sizable cohort exceeding 300 patients. Remarkably, it is the first study examining growth factor and drug‐induced pneumonitis incidence in FL patients. The results, independent of diagnosis, hold relevance in treatment decision‐making concerning G‐CSF use.

The weakness of this study was a retrospective study design. Despite the moderately high number of patients, the number of DIP cases was limited leaving room for a chance to affect the results. Also, in this study comparison between G‐CSF with single administration and daily administered G‐CSF cannot be made due to small amount of pneumonitis cases. If long‐lasting G‐CSF increases the pneumonitis risk more remains unclear.

Recognition of possible DIP is crucial in evaluating lymphoma patients with respiratory symptoms, particularly in the presence of G‐CSF use. Accurate differential diagnosis, distinguishing pneumonitis from bacterial pneumonia and pneumocystis lung infection is important, also in order to reduce antibiotic resistance. G‐CSF should be administered exclusively when substantiated by indications. Naturally, further studies are required.

## AUTHOR CONTRIBUTIONS

**Elina Kaprio:** Formal analysis (lead); investigation (lead); writing – original draft (lead). **Roosa Prusila:** Data curation (lead). **Susanna Tokola:** Data curation (equal). **Milla E. L. Kuusisto:** Data curation (equal). **Esa Jantunen:** Data curation (supporting). **Hanne Kuitunen:** Funding acquisition (supporting). **Taina Turpeenniemi‐Hujanen:** Supervision (supporting); writing – review and editing (supporting). **Outi Kuittinen:** Project administration (equal); supervision (lead); writing – review and editing (lead).

## CONFLICT OF INTEREST STATEMENT

No potential conflict of interest was reported by the authors.

## Data Availability

The data that support the findings of this study are available on request from the corresponding author. The data are not publicly available due to privacy or ethical restrictions.
